# Embracing Additive Manufacturing Technology through Fused Filament Fabrication for Antimicrobial with Enhanced Formulated Materials

**DOI:** 10.3390/polym13091523

**Published:** 2021-05-09

**Authors:** Waleed Ahmed, Sidra Siraj, Ali H. Al-Marzouqi

**Affiliations:** 1 Engineering Requirements Unit, College of Engineering, United Arab Emirates University, Al Ain 15551, United Arab Emirates; 2 Chemical Engineering Department, COE, United Arab Emirates University, Al Ain 15551, United Arab Emirates; sidra.siraj@uaeu.ac.ae (S.S.); hassana@uaeu.ac.ae (A.H.A.-M.)

**Keywords:** antimicrobial, antibacterial, 3D printing, fused filament fabrication, composite material

## Abstract

Antimicrobial materials produced by 3D Printing technology are very beneficial, especially for biomedical applications. Antimicrobial surfaces specifically with enhanced antibacterial property have been prepared using several quaternary salt-based agents, such as quaternary ammonium salts and metallic nanoparticles (NPs), such as copper and zinc, which are incorporated into a polymeric matrix mainly through copolymerization grafting and ionic exchange. This review compared different materials for their effectiveness in providing antimicrobial properties on surfaces. This study will help researchers choose the most suitable method of developing antimicrobial surfaces with the highest efficiency, which can be applied to develop products compatible with 3D Printing Technology.

## 1. Introduction

3D Printing, also known as fused filament fabrication (FFF), continues to open new routes to the production of high-performance and complex structures with enhanced properties and dynamic shapes that are unattainable via conventional fabrication methods. Developing new material for different applications of the 3D Printing technology [1] usually involves many challenges, such as the mechanical properties [2] as well as the failure mechanism [3,4,5]. The FFF technology opened new horizons for a wide range of applications [1,2] but limitations still face this fast-moving sector [6,7,8]. Although the 3D Printing technology contributes to the increasing levels of wasted material of polymeric base [9,10], on the contrary, there are unlimited benefits that are continuously increasing day by day. Owing to its innovation-driven approach, 3D Printing has been expanding to several fields, such as analytical chemistry for chromatography [3] and fluorescence techniques [4], optic fibers for increased optical and mechanical performance [5,6], membrane technology for increased adsorption of chemicals [7], space technology to develop tools on-site [8], and medicine for the development of implants and medical devices [3,5,7,9,10,11,12,13].

The ease of modeling and experimentation helps with progressive growth and expansion of knowledge in these fields, which can be of significant value to clinical areas. 3D Printing has been extensively employed in the biomedical field, especially in biomodelling, for the fabrication of scaffolds and implants, such as for dental restorations and tissue engineering, bone and fracture healing [1,14] and even for drug delivery because it is highly flexible and faster than current methods such as machining [15,16]. Moreover, the positive effects on the mechanical and biological properties of designed microporous scaffolds manufactured by commercial 3D Printing technology promote the use of 3D printed scaffolds as viable candidates for further research for clinical applications [16,17]. 3D Printing is also a highly cost-effective approach, cutting costs and improving economic efficiency.

**Figure 1:**
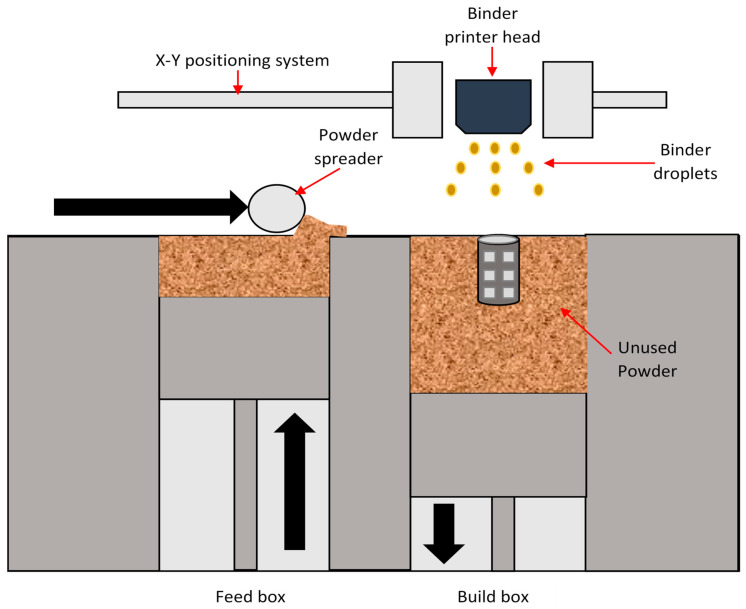
Schematic representation of FFF.

Several techniques that have been commonly applied worldwide for 3D Printing include FFF, selective laser sintering (SLS), digital light processing (DLP), and stereolithography (SLA) [13,18,19,20]. Of these, FFF has been extensively used to develop medical devices. In FFF, the feedstock to the 3D printer is a simple filament made from a thermoplastic material that is heated to soften and extrude through a printer head or nozzle, which builds layers driven in an X–Y orientation. FFF has been reported to be an excellent technique to fabricate small mechanical parts, providing sufficient precision, which allows rapid modifications to be made during the process itself. A simple FFF schematic is shown in Figure 1 [18].

With the use of 3D Printing increasing in biomedical applications, the surface properties of the developed 3D printed material are essential because this material will be in direct contact with the human body. Common microorganisms that are highly associated with infections include Staphylococcus spp. strains, such as Staphylococcus epidermidis [21], and Gram-negative Enterobacteriaceae strains, such as *Escherichia coli* (*E. coli*) [22]. The adhesion of bacteria to the surface is facilitated by several factors, such as hydrophobicity and surface tension, which is followed by accumulation resulting in a biofilm [23]; however, it may not always be harmful [24]. Thus, having an active antibacterial surface on the 3D-printed material is of vital necessity to prevent any microorganisms from developing on human tissues and infecting the human body. Even though conventional approaches involve the release of metal ions, such as fluoride, silver, or zinc, to prevent and fight bacterial infections, this method still results in decreased mechanical properties, causing toxicity to surround tissues. Additionally, the fabrication of antimicrobial materials is preferred for coating the surfaces of materials, even though this adds an additional process, costing time and money in any clinical field [25]. 

Polymer composite materials have gained the attention of researchers worldwide in a variety of fields, from printing and packing applications to several other applications [17,26,27,28]. Due to filler addition, significantly enhanced properties, such as mechanical and thermal properties and conductivity, are attained; thus, increasingly, applications prefer the use of polymer composite materials instead of pure polymers. One of the most promising techniques that has been used is the addition of metallic particles as fillers to provide enhanced antimicrobial and antiviral properties to the commonly developed polymer composite materials being used in several applications. Currently, viral transmission through either touch or contaminated liquids, such as blood, poses a massive threat to populations worldwide. The issue of the inactivation of viruses in media requires an upfront solution, and the development of novel means to inactivate viruses is highly desirable. One of the most novel developed strategies is to incorporate copper nanoparticles (NPs) into the matrix. Copper has potent virucidal properties, and copper’s neutralization of infectious bronchitis virus, poliovirus, human immunodeficiency virus type 1 (HIV-1), and other enveloped or nonenveloped single- or double-stranded DNA or RNA viruses has been well reported [29]. The ability of free copper ions in copper NPs to break the membranes of microorganisms alters their DNA, thus eliminating them, which confirms their great potential for keeping material surfaces free of pathogens. Based on the broad-spectrum antiviral effectiveness of copper, the incorporation of copper NPs in any intended hybrid composite material could inherently add significant value to the final product, especially with regard to the COVID-19 pandemic situation [30]. 

Another metal that has caught the attention of researchers worldwide is silica. Due to its high chemical and thermal stability and excellent antimicrobial properties, silica has been used in several applications to provide enhanced features to composites. For instance, for the treatment of bacteria, silver NPs are widely used in several industries. The main challenges in the use of silver NPs is to provide an antimicrobial specifically for antibacterial surface suitable for a wide range of substrates with mechanical and thermal resistance with prolonged antibacterial properties and that is suitable for wide temperature ranges. Moreover, the agglomeration of silver NPs has always remained a challenge because it decreases their antiviral effects. A study reported excellent antiviral capabilities for a hybrid material against two model viruses for different water conditions. silver NPs incorporated with SiO_2_ showed minimized agglomeration and minimal particle release in water environments [31].

Another study reported the production of a series of mesoporous silica nanoparticles (MSNPs) using room-temperature ionic liquids (RTILs) to study the mass-transport properties by investigating the controlled release profiles of these materials and utilizing the RTIL templates as antibacterial agents. The study showed that the antibacterial activity was dependent on the rate of diffusional release of the pore-encapsulated RTIL, which was in turn governed by the particle and pore morphology of the MSN materials, thus showing the good potential for silica-based mesoporous materials to be used in several applications that require controlled release delivery [32]. The literature also supports the idea of the production of antibacterial silver nanocluster–silica composite coatings by radiofrequency co-sputtering techniques, which can result in peculiar antibacterial properties [33]. Additionally, another method by which silica is gaining popularity is allowing monomers to be attached to its surface, providing enhanced properties. For instance, silica particles were used to treat water, and the results supported excellent biocidal efficacy against bacteria, thus showing their good potential to combat microorganisms in water [34,35]. Thus, the use of silica-based composite materials with added antimicrobial and antiviral properties enhanced with copper NPs can open pathways for its significant use in fields that require high-purity surfaces, such as medicine, food packing, and space industries [2,10,23].

Additionally, polysaccharides are now also considered to develop material for antimicrobial materials. Bacteria is a large reservoir for the development of polysaccharides. These materials have exhibited antibacterial property by formulating films which have shown broad anti-bacterial activity [36]. Since these bacterial polysaccharides can follow an ordered structure, it is seen as an excellent bioprinting material to provide antibacterial effect. Commonly used bionics are alginate, bacterial cellulose and hyaluronic acids [37]. 

Antimicrobial mechanisms in 3D printed material simply by exploiting the antimicrobial property of the incorporated NPs. NPs are usually synthesized following up physio-chemical processes such as using solvents and through reactions such as reduction and oxidation. However, a lot of green/bio NPs are also considered to reduce the adverse effects of toxic solvents and harmful by-products produced [38].

**Figure 2:**
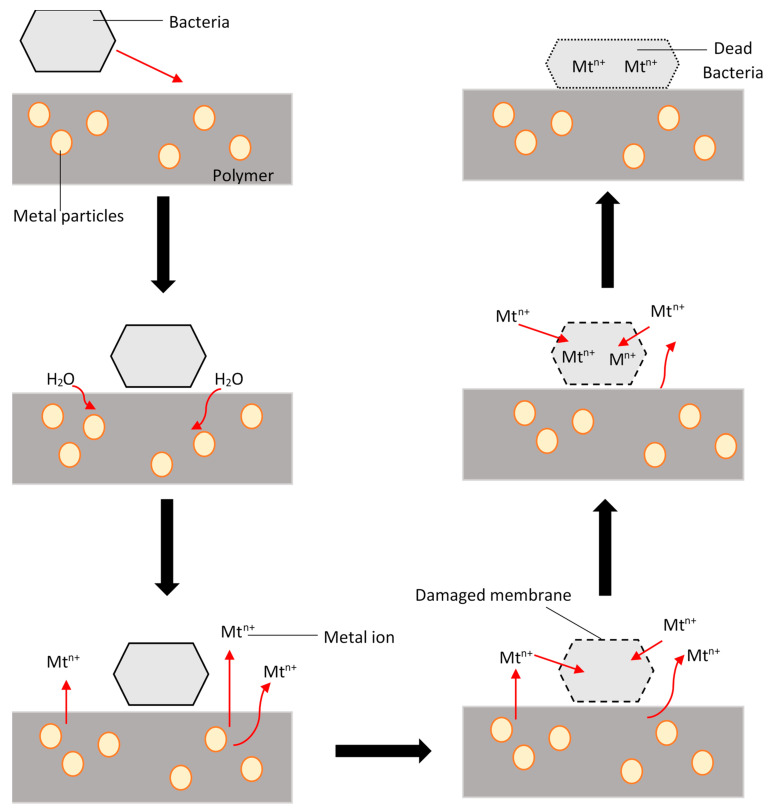
Mechanism of the antibacterial behavior of polymer composites with metal ions [39].

Furthermore, to fully understand the mechanism of how metal ions produce this antimicrobial effect for instance against bacteria, a simple representation of how these metallic particles at a micro or nanoscale are illustrated in Figure 2 for a polymer/composite material. Initially, the adsorption of bacteria on the surface of the polymer triggers the diffusion of water through the matrix. Next, the water, along with dissolved oxygen, reaches the surface of the metal particles, which allows the dissolution process. This leads to the formation of metal ions. Consequently, the metal ions deposit on the surface of the composite and damage the membrane of the bacteria, which results in the metal ions diffusing into the bacteria. The same can be applied for any polymer/metal material with a biocide agent that is embedded in the matrix [39].

Since metallic ions have been extensively used to provide antipathogenic effects, the combination of 3D Printing alongside metallic particles can result in successful developments of antimicrobial materials, tools, and surfaces [40]. Li et al. [19] successfully prepared an antimicrobial photosensitive resin using the DLP technique with active bacterial resistance. Yue et al. [41] also successfully developed an antimicrobial composite FFF that was positively grafted with metal ions capable of killing any bacteria contacting the surface using SLA. Another study conducted by Muwaffak et al. [42] used hot-melt extrusion to integrate metal ions into a polymer-based filament, which was then used to 3D print a wound dressing. Gutierrez et al. [15] followed a more natural approach by developing antimicrobial alginate hydrogels that were used to 3D print. Ben et al. [29] used surface-modified β-tri-calcium phosphate (β-TCP) granules for 3D Printing for bone tissue engineering. In another study conducted by Huang et al., polymer/ceramic blends prepared with the FFF technique to develop bone scaffolds were characterized [43].

In particular, the use of antimicrobial composite material along with FFF specifically for bio-applications has been widely studied [44,45]. For instance, biodegradable polymers such polylactic acid- (PLA) based polymers are often used to develop filament that possess antibacterial properties using FFF as discussed by Bronstein et al. [46]. Moreover, the antibacterial property is added with the help of processing techniques such as co-extrusion with particles for instance using chitosan as studied by Mania et al. [18] or using metallic nanoparticles as done by Rezić et al. [47]. Biomedical applications such as dental resins as studied by Bayarsaikhan et al. [48] and drug delivery as highlighted by Shaqour et al. [49,50] highly promote a practical approach of using 3D Printing along with compatible antibacterial material. Bartolomé et al. [51] and Navaruckiene et al. [52] both emphasize the use of FFF as a promising approach for bio fabrication of antibacterial material and suggest that using metallic particles alongside biodegradable polymers show great compatibility to the human body and is expected to be considered as the more superior and dynamic approach of development in the field in the upcoming years.

Thus, several metal ions have been incorporated to prepare antibacterial materials compatible with 3D Printing technology, which can serve as a cost-effective approach, especially in the field of biomedicine, which requires high purity and highly anti-pathogen surface materials. 3D Printing can offer a higher precision compared to conventional methods that can cater to more customizable solutions that are increasingly being desired. This article provides a comprehensive review of the development of new antibacterial material and their use in 3D Printing technology. The potential application of using metallic fillers as antibacterial materials in 3D Printing technology in several other fields will also be highlighted. 

## 2. Techniques and Effectiveness of Developed Antibacterial 3D-Printed Materials

To obtain a clear understanding of whether antibacterial materials are providing good effectiveness against bacteria development on surfaces, selected recent studies (2015–2020) on developing antibacterial materials for 3D Printing were compared. Standard laboratory procedures for ISO 22,196 were followed to test the antibacterial properties in all studies. The tests were mainly conducted against methicillin-resistant *Staphylococcus aureus* (*S. aureus*), standard *Staphylococcus aureus* (*S. aureus*), and *Escherichia coli* (*E. coli*) because these are familiar strains known to cause infections at homes and hospitals.

One of the biggest challenges of medical implants is the ability to consistently inhibit bacterial adhesion and infection. Alternate materials to antibiotics are now being explored, particularly carbon-derived materials, such as graphene-based materials (GBMs), and they have started to receive increased attention owing to their potential antibacterial properties. Melo et al. [53] incorporated graphene oxide (GO) to develop a polycaprolactone (PCL) fibrous-based scaffold because smaller oxidized versions of GBM’s have been reported to have a higher biocompatibility with cells. GO was dispersed in PCL using suitable solvents. After attaining optimum concentrations, these formulations were extruded through a needle for scaffold plotting. The time-dependent bacterial effect was studied, and a significant increase in the bacterial death rate from <20% in neat PCL up to 80% in the GO/PCL composite material was observed. The results also revealed that the composite material allowed human cell activity to show fibroblast adhesion, spreading, and colonization. The results showed the potential of GO concerning its antibacterial properties in scaffolds that could be used in medical implants and devices.

Photosensitive resins with antibacterial properties are vital, especially for biomedical applications. Studies have reported that methyl acrylates are widely used in DLP 3D Printing because they cure fast and result in a rich variety of products. Moreover, thiol-ene-based systems can accelerate the printing speed and use a lower amount of photoinitiator, resulting in reduced costs and lower yellowing of the resin. They are also employed in DLP 3D Printing techniques that require increased resolution and higher accuracy. In a study conducted by Li et al. [19], a ternary, thiol-ene-acrylate, which has a high photopolymerization rate, was selected as the 3D Printing matrix material owing to its reduced shrinkage property, which can result in increased 3D Printing accuracy. The study incorporated two quaternary ammonium salt-based antibacterial agents, QAC and SH-QAC (ammonium salt with added thiol group), which were prepared via dissolution in solvents, to provide the desired antibacterial effect. The QAC group was added to the matrix via in situ copolymerization, and SH-QAC was added to further increase the compatibility between the matrix and the QAC group. It have been reported that a 100% antibacterial rate was achieved with 4 wt% QAC and 10 wt% SH-QAC. However, the antibacterial effect of the QAC was superior to that of SH-QAC with the same amount. This could be because SH-QAC has long chain segments and the antibacterial group is possibly immobilized inside the resin after the photocuring process. Both antibacterial photosensitive resins were used to develop a 3D-printed tooth model, which showed high precision, suggesting that this technique is feasible for biomedical applications. 

Antibacterial resins are commonly used in dental applications to deposit antibacterial agents, such as fluoride, zinc ions, and antimicrobial peptides into resins [54]. Additionally, silver ions have been reported to be able to kill bacteria, then dissociate from dead bacteria, and effectively repeat the sterilization process. Silver-based salts or layered inorganic fillers, such as zeolites, are commonly used to develop silver-based inorganic antibacterial agents via ion-exchange or chemical deposition techniques. Moreover, nanomaterials, such as halloysite nanotubes, which have a high aspect ratio and large volume, are often used with silver ions to provide increased antibacterial effects for a long duration. In a study conducted by Sa et al. [55], DLP-3D-printed stereolithographic resins (SLR) with silver halloysite (Ag-HNT) were prepared to study the antibacterial effect against Streptococcus mutans, which is a main reason for dental caries. Urethane dimethacrylate (UDMA) was used as the main component, and triethylene glycol dimethacrylate (TEGDMA) was used as a diluent to decrease the viscosity of the resin fluid; a photosensitizer was also added. The material exhibited strong antibacterial property up to 99% even after repeated extraction for over 1 week. This prolonged antibacterial effect is highly desired in dental applications because saliva cannot be easily replaced in the retention area, promoting Ag-HNT/SLR antibacterial material as having good potential for use in dental applications. 

Biomedical applications have also began utilizing biopolymer-based hydrogels or salt-hydrogels owing to their excellent biocompatibility and relatively low cytotoxicity [56,57]. This is a result of their structural similarity to the soft tissues and surrounding cell matrix and due to their high water content in their structure. Moreover, because hydrogels provide a moist environment, they are believed to promote wound healing. Alginate-based hydrogels are widely accepted as good biopolymer hydrogels. They can be easily ionically cross-linked with divalent ions owing to the presence of the negatively charged carboxylic acid group. The ionic cross-linking is a relatively fast process that specifically results in the incorporation of alginates in the 3D Printing technique for artificial implants and biomedical devices. In a study conducted by Guiterrez et al. [15], alginate beads were developed by ionic cross-linking an aqueous alginate solution at 1 wt%. Once the beads were prepared, an infusion pump was used to drop a copper nitrate solution into the bead solution for the ion-exchange process. Bacterial cellulose was used as a rheological modifier in this case with the fermentation of Gluconacetobacter xylinus. During extrusion, the alginate was ionically cross-linked by an ionotropic process with 0.1 M CaCl_2_ or Cu(NO_3_)_2_ solutions. Then, the 3D-printed scaffolds with a 4 wt% alginate concentration were tested against *S. aureus* and *E. coli*. The tests showed a distinct inhibition halo independent of the bacteria strain with a 50-mM copper salt concentration. The results were similar to those in the literature in terms of the obtained biocidal effect but without a specific effectiveness percent reported.

A study led by Zuniga J. [58] focused on developing a low-cost upper limb prosthesis for the human finger, and shoulder prosthesis [59] using 3D Printing technology because using a customized prosthesis can help improve bi-manual activities and unilateral activities in terms of the functions of grasping and holding. For the developed shoulder prosthesis, manually adjustable options increased the wrist movements, elbow extensions, and shoulder rotations. For this study, PLACTIVE™ with 1% antibacterial nanoparticle additive was purchased as the ready-made 3D Printing material. PLACTIVE™ is a pure high-grade type of polylactic acid (PLA), which has copper NPs incorporated as the key material that provides the antibacterial effect. Copper NPs are highly effective at killing bacteria as well as other pathogens, including fungi and viruses. The customized prosthesis was designed and developed using extrusion and FFF. The results showed that the surface of the developed prosthesis was effective against 99.99% to *S. aureus* and *E. coli*. The study also stated that the antibacterial properties of the 3D Printing PLA filament were not altered after addition of the NPs or after the extrusion, which supports during the positive post-processing modifications needed for customization of any type of prostheses. From this study, they found that using PLACTIVE™ in 3D Printing technology could provide a simple inexpensive method to develop customizable patient-specific prostheses and potentially medical devices.

As discussed earlier, metals, such as zinc, copper, and silver, are extensively used to provide antibacterial properties to pure polymeric matrices. In a study conducted by Muwaffak et al. [42], polycaprolactone (PCL) was used to produce filaments for 3D Printing to develop patient-specific wound healing dressings. Wound dressings are used to provide protection from contamination and can be used to deliver topical bioactive agents in the form of ointments and creams to aid faster healing. In that study, silver-loaded filaments, copper-loaded filaments, and zinc-loaded filaments were prepared using dissolution in suitable solvents and PCL. The obtained filaments were chopped and sent into a hot-melt extruder to increase the homogeneity between the polymer matrix and filler content. The study reported Ag-PCL and Cu-PCL having the highest bactericidal effects against the *S. aureus* bacteria strain with increasing amounts resulting in a more potent inhibition compared to Zn-PCL. As 3D scanning was used to develop the patient-specific 3D model for wound dressing, this approach is a novel technique to tailor patient-specific antibacterial wound dressings.

Dental restorations with potential antibacterial properties have been one of the leading branches of biomedicine that has incorporated 3D Printing technology. In a study conducted by Yue et al. [41], positively charged quaternary ammonium-based salts were grafted onto the surface of a material to kill bacteria on contact. These charged groups interact with the cell wall of the bacteria and break their lipid membrane, releasing cytoplasmic elements. The frame component was chosen to be diurethane metacrylate (UDMA) because it can be rapidly cross-linked, along with camphorquinone (CQ) as the photosensitizer, ethyl 4-(dimethylamino)benzoate (EDMAB) as the co-initiator, and glycerol dimethacrylate (GDMA) as a diluent to decrease the viscosity. The antibacterial agent, which was the quaternary ammonium-based salt, was varied according to the alkyl chains from n = 2 to n = 12 (QA_Cn). The quaternary ammoniums groups were covalently added into the matrix by in situ copolymerization with the resin. FFF was used to 3D print a tooth model wherein all the layers fused well during photocuring. The antibacterial efficacy of the surface of the tooth model was observed before and after coating with a salivary conditioning film (pQA_C12). The results showed almost no presence of bacteria with increasing chain length. Moreover, incorporation of the QA_C12 within the polymer resulted in less bacteria contact killing than the copolymerized QA_C12 that was directly present in the resin. However, the use of antimicrobial resins is a promising approach for several dental applications, such as adhesives, cement, or dental restorations, with the use of high alkyl chain ammonium salts.

Table 1 shows a comprehensive comparison of selected parameters of the above-mentioned studies in terms of polymers chosen, particles, and chemical agents used to provide the antibacterial effect. Table 2 compares the 3D Printing parameters and tests done to develop the antibacterial materials. Additionally, a summary of the selected studies is shown in Table 3 along with the particles used, their antibacterial efficacy, and the chosen application.

## 3. Applications Using 3D-Printed Antibacterial Materials 

### 3.1. Biomedical Field

Over the last 20 years, the drawbacks of current materials used in the medical field, mainly for bone applications, have led advances in the area, particularly toward developing a synthetic bone material alternative. Owing to the ease of experimentation, prototyping, and customized tailored output of 3D Printing, its use has significantly increased in bone, dental, and tissue restorations. 3D Printing has resulted in materials that are biocompatible, osteoconductive, and can result in the desired porous structure that is fully compatible with the growth of the surrounding capillaries and promotes drug delivery. The aim of any alternative bone material or scaffold is to provide mechanical support needed and over a prolonged time to become resorbed as new bone and aid the growth of surrounding tissues [16].

In a study by Roopavath et al. [60], hydroxyapatite (HA) scaffolds were printed using extrusion-based 3D Printing for bone grafting applications. The results indicated that the porosity and mechanical properties of the printed scaffolds could be controlled using 3D Printing technology. As the scaffolds can be printed on models based on computed tomography, the accuracy and precision of this approach inevitably depict it as a promising technique for bone implant developments. Recently, the FFF approach was tested with an applied ceramic coating of silicon nitride (Si_3_N_4_) on biomedical grade commercially available pure titanium (cp-Ti) via the laser sintering process. The Si_3_N_4_ coating was able to provide both the antibacterial and osteogenic properties of the bulk material to the cp-Ti substrate, resulting in properties that match current biomedical implants [61].

As discussed earlier, studies have reported copper compounds that have high antipathogenic properties; thus, their use in the medical field is quite extensive. As they can aid in developing low-cost medical devices with high antibacterial properties, copper particles are excellent alternatives to other compounds, such as silver, which in some cases has been reported to cause skin irritations [42]. Several studies on 3D-printed medical prostheses for the upper limbs, including arms, shoulders, and fingers, have stated the capability of 3D Printing to provide low-cost, customizable solutions with the possibility of incorporating the required antibacterial surfaces [59,62,63]. 

3D Printing is widely used in dental applications. In a study conducted by Yamada et al. [64], silver compounds were incorporated to develop dental prosthesis to provide an antibacterial effect to the surroundings, mainly preventing caries. Silver compounds were also studied by Sa et al. for the preparation of dental restorations with excellent antibacterial effects [55].

Wound dressing applications have also resulted from the 3D Printing approach. The use of a wound dressing will help isolate the injury and external surroundings and aid in faster healing by reducing tissue edema and promoting hemostasis. Topical agents combined with wound dressings are used to provide increased wound healing and to treat any buildup of local infections by minimizing their residence times [65,66]. A 3D-printed chitosan-pectin (CS-PEC) biopolymeric hydrogel wound dressing that incorporated lidocaine hydrochloride (LDC) has been studied [67]. These hydrogels were developed using cross-linking of CS and PEC, and the scaffolds were 3D printed using extrusion-based 3D Printing. The wound dressing exhibited good physical integrity, flexibility, and skin adhesion. Constant drug release was also observed, supporting the applicability of 3D Printing for use in wound dressing applications. Another study conducted by Muwaffak et al. involved 3D scanning to develop 3D models to customize the shape and size of a wound dressing. The extrusion of PCL pellets combined with zinc, copper, and silver metal particles of different loadings was developed. The results of the antibacterial property against the common *S. aureus* strain of bacteria of the developed wound dressing suggested that silver and copper wound dressings resulted in the most potent antibacterial properties. This simple 3D Printing customizable approach has excellent potential for developing wound dressings with added antimicrobial properties.

### 3.2. Use of Composite Materials in 3D Printing for Material Chemistry

As mentioned earlier, AM can deliver high-performance and multifaceted structures with improved properties that are unattainable by current conventional fabrication methods. The use of ceramics and fillers, such as silica, aluminum, and zirconium, are leading to rising developments in the field of advanced materials owing to their high thermal stability and high stability. Powdered ceramics have been widely incorporated into 3D Printing to provide enhanced properties, such as increased strength and porosity to the developed material. Several examples of 3D Printing technology in the field of material science and chemistry have been studied, such as in mass spectrophotometric techniques and fluorescence techniques [4,68]. The results support FFF, providing an excellent method to fabricate small mechanical parts with sufficient precision, which allows rapid modifications to be made during the process itself. A study focused on the modification of a low-cost open-source 3D printer into a 3D silica gel layer printer, which can be applied to thin layer chromatography. Using this technique, the potential of 3D Printing of adsorbents for analytical chemistry was studied. The results supported the fast, inexpensive production of the layers, opening new pathways for novel planar chromatographic separations [3]. A study was conducted to develop a novel silica–titania-based 3D-printed material, resulting in increased use of optical performance materials [69]. 

Another study also reported the production of high-quality fused silica glass by 3D Printing using a silica nanocomposite [70]. In that study, the ability to develop fused silica glass using silica nanocomposites showed the ability to produce complex shapes and structures on a macro and micro scale using 3D Printing technology. 3D-printed ceramic-based scaffolds have also gained wide interest in the medical industry [16,71]. For instance, the effects of the mechanical and biological properties of designed microporous scaffolds manufactured by commercial 3D Printing technology have been studied. The results showed the use of silica-based 3D-printed scaffolds as viable candidates for further research for clinical applications [16,17]. 

Another study by Li et al. reported the effects of Al_2_O_3_ dispersed in a UV-curable acrylic-based resin, which may be compatible with SLA-3D Printing of micro components with complex shapes [46]. Yang et al. reported a 3D Printing gel printing process that was based on a water-based gelation system that incorporates zirconia, which can be applied to develop complex parts for magnetic applications [72]. Rapid advancements in this field are expected owing to the ease of incorporating ceramic powders with polymers and their ability to control and vary the desired properties of the developed material. 

### 3.3. Use of Metallic Fillers in Composites in 3D Printing Membrane Technology

The increased use of metallic fillers in composite materials has recently gained huge interest in the water treatment sectors to remove waste compounds or heavy metals owing to their increased chemical and thermal stability, higher permeation rate, and increased selectivity compared to polymeric sheet membranes. However, owing to the high costs involved in developing larger quantities of ceramic–polymer composite materials, there is a need to shift toward a cost-effective approach to develop them commercially that can be easily applied industrially, which could potentially be accomplished via 3D Printing to provide good antipathogenic surfaces. 

Several techniques have been utilized to develop polymer composite materials such as spray-coating, spin-coating, and dip-coating via nano-filtration and ultra-filtration (UF). UF has also been extensively studied to treat wastewater because it can remove heavy colloidal substances; however, studies have shown that it does not have the ability to remove 1% oil by volume because the sizes of the oil droplets are below 10 µm [73,74]. Moreover, polyvinyl alcohol (PVA) and cellulose acetate as membranes have also been studied owing to their anti-fouling properties and biodegradability [75]. 

Recently, 3D Printing technology using ceramics and clays has also gained wide interest. Metal–organic frameworks (MOFs) are commonly used as adsorbing materials. SLS to print highly porous flow-through filters containing the MOF copper (II) benzene-1,3,5-tricarboxylate (HKUST-1) has been studied. The printing did not have any negative impact on the structure of the MOF, thus supporting the use of 3D Printing technology for filter use [13]. Inject 3D Printing has also been employed in another case of ceramic filters for water treatment using clay as the raw material. That study reported that inkjet 3D Printing is an effective technology for forming clay ceramics that have the potential for use as microfiltration membranes, for example, as pre-filtration media to improve water quality. Another study also reported the use of clay to develop filter material using 3D Printing because it is a cost-effective approach for treating waste from water. The study showed that excellent filtration was achieved, thus increasing the water quality [7]. Additionally, as metallic particles are proven to provide high antibacterial efficacy, there may be the added benefit of killing bacteria and treating water.

In another study, microporous silica-based composite material was developed to study the effective removal of Sr-90 and Cs-137 (both are high heat emitting nuclides) from acidic high-level liquid waste, which is produced in the reprocessing of nuclear spent fuel. As these ions can be used for energy generation, it is vital to study the effective removal of such nuclides [76]. Despite the challenges involved in the removal of such highly beneficial ions, silica has been used as a desirable filler in a polymer composite material owing to its high thermal stability and selectivity to Sr-90, thus showing the versatility of its application for treating liquid wastes. Another study highlighted the ability of a silica-based composite material to recover gold and palladium from AuCl_3_ and AgCl_2_ in acidic aqueous solutions [77]. Moreover, polymer/silica/amine fibers have been reported to show improved adsorption of CO_2_ from flue gas compared with currently used functional fibers [78]. Thus, silica-based polymer composites have been of significant interest because they have the capability to recover precious metals, remove toxic materials, and be incorporated with 3D Printing. Silica has also been proven to provide added antibacterial properties, which can be very useful for water treatment.

### 3.4. Space Applications

3D Printing has recently also been accepted for use in space applications. 3D Printing in the consistent microgravity environment of the International Space Station (ISS) can be used to develop parts made from particular materials needed in space. For example, the first 3D-printed tool on the ISS was a wrench built using FFF by depositing 104 layers of plastic [79]. The ability to manufacture 3D-printed parts and tools on demand will dramatically reduce the time it takes to get parts to orbit and increase the reliability and safety of space missions while reducing costs. Current space missions take months to years to get parts to orbit [8]. Moreover, FFF with extrusion-based machines functions similarly in microgravity as it does on the ground, allowing for a fool-proof concept of 3D Printing in space [10].

The extrusion-based fused filament fabrication method using thermoplastics represents a robust and straightforward methodology that is applicable to printing parts for both current and future human spaceflight exploration missions [80]. Literature reports have indicated that polymeric-based materials have potential as space-capable materials because they can produce extravehicular activity, repair tools, and even satellite structures that can be created on-site and on-demand. This will enable safer, less mass-intensive missions and scientific experiments, provide an advantage to FFF [81]. Among the most commonly used thermoplastic polymer materials are acrylonitrile butadiene styrene and PLA because these materials have been reported to maintain their shape at cabin temperatures [80]. 

According to the database maintained by NASA on the ISS, 17% of the total spare parts are made of metallic materials. It has been reported that powder bed fusion and, in particular, laser beam melting (LBM) are the preferred FFF technologies for commercially producing ready-to-use high-performance metallic parts with desired size specifications [82]. A stainless steel metal powder was successfully processed in the LBM process under microgravity (μ-g) conditions in a parabolic flight campaign, which showed no significant deviations from a part manufactured at 1 g, thus supporting the feasibility of the LBM process for 3D Printing ready-to-use metal parts in space. 

3D Printing systems have been advancing in the medical field as well. A bioprinting system for the ISS has already been developed. This technique allows 3D Printing of thick tissue and organs using adult stem cells, supporting that 3D bioprinting can result in exact movements and control of the final dispensation [83]. Advances in the medical field could be a great breakthrough for the space industry. The literature also shows that NPs of various metals and metal oxides, such as zinc oxide nanoparticles (ZnONPs), cuprous oxide nanoparticles (CuONPs), silver nanoparticles (AgNPs), copper (I) iodide nanoparticles (CuINPs), gold nanoparticles on silica nanoparticles (Au-SiO2NPs) and some quaternary ammonium cations, which are commonly called QUATs, are very promising for inactivating viruses. The increasing use of polymer composite materials worldwide in a variety of fields promotes filler addition (whether metallic or non-metallic), which significantly enhances the properties such as mechanical, thermal, and conductivity properties [17,26,27,28]. One of the most novel strategies developed is the incorporation of copper NPs into the polymer matrix. Copper has strong virucidal properties, and copper’s neutralization of infectious bronchitis virus, poliovirus, human immunodeficiency virus type 1 (HIV-1), and other enveloped or nonenveloped single- or double-stranded DNA or RNA viruses has been well reported [29]. Based on the wide range antiviral potency of copper, the incorporation of copper NPs in an intended composite material could inherently add significant value to the final 3D-printed product, especially with regard to providing antipathogenic surfaces, such as medical equipment that requires high purity for use by astronauts in a safer and more efficient way. Additionally, another mineral that has caught the attention of researchers worldwide is silica. Owing to its high chemical and thermal stability and good antimicrobial properties, silica has been used in several applications to provide enhanced properties to composite materials, thus making it a good candidate for the final 3D-printed product. 

Investigations into the properties of regolith materials [84] (material deposited on the soils of planets) are also being conducted via powder bed fusion-based 3D Printing, which show the recent advancements in this field. Nevertheless, developing such particulate matter is not easy or efficient. Perhaps already developed materials, such as composite materials, would provide a better alternative to investigate further. However, owing to the high costs involved to develop larger quantities of polymer composite material, there is a need to shift toward a cost-effective approach to develop them commercially, which can be easily applied industrially and could potentially be advanced using 3D Printing. 

Moreover, because thermoplastics have the ability to be recycled as feedstock and then printed again, using recycled materials as printer feedstocks could be a viable approach. One goal is to utilize non-metallic composite materials (recycled fiber-based composite materials) that could provide an effective long-term solution to the buildup of trash in space and its effective disposal [85]. Currently, the in-space manufacturing program conducted by NASA is focused on evolving manufacturing technologies from Earth-reliant to Earth-independent technologies. This is directed toward future hardware fabrication via FFF, which includes feedstock recycling, the development of parts, printable electronics, and investigations into additive manufacturing of metallics and external repairs [80,86].

Furthermore, it is noteworthy to state that due to the sudden rise in the COVID-19 pandemic, extensive researches worldwide have been conducted focusing on the production and development of antimicrobial material just in the span of a year [87,88,89,90]. More and more focus has been provided on the fabrication of antimicrobial surfaces with the incorporation of polymeric base along with additives [18,46,91,92,93]. The positive results from such antimicrobial studies have been promising and are providing hope to fight against such potent diseases. 

## 4. Gaps, Challenges, Opportunities and Future Trends

The ease of customization of devices which can be developed with the minimum processing time makes this technology the preferred alternative for tailoring specific shapes and tools [54]. Moreover, since polymer science is bound to advance in the medical field, the rapid prototyping approach allowed by the 3D Printing technology results in control over the developed product with the desired shape and properties [94]. However, one of the critical challenges of 3D Printing is the alignment of the composite fibers used. Research is underway to improve this. One of the solutions developed to tackle this issue is using continuous fiber-reinforced with thermoplastics, which can be implemented for large-scale industrial independent processes [95,96]. Another challenge is that the influence of parameters can be material dependent, and this interconnection requires further investigation to fully develop complex materials via 3D Printing. 3D Printing of antibacterial substances is still a relatively new field, and there is still a lack of a database on modeling of this method. However, this technique has considerable potential to analyze composite structures and further improve enhancements of 3D Printing technology with slight modifications. Complex fields, such as membrane technology and space technology, that incorporate 3D Printing with the addition of antibacterial materials to provide high-purity tools and devices promote the use of 3D Printing and its increasing potential and reliability for such fields in the future. Moreover, adopting antimicrobial material through 3D Printing technology opens the doors for a wide range of applications, especially the medical sector, such as producing assistive devices [97,98]. In addition, due to the COVID-19 pandemic crisis, the antimicrobial material can be used to fight the spread of the virus of the possible contaminated buttons instead of replacing the exist system [99]. Moreover, the FFF is an excellent, affordable, traditional technology [100] that can be incorporated with antimicrobial material with different natural material to produce different engineering components [101,102]. Eventually, using antimicrobial material through the 3D printing technology must be evaluated [103] to predict the final product properties [104] as well as to explore the possibilities of the failure while being in service [105] through experimental investigation, and even could be achieved theoretically through simulation [106], especially we are dealing with composite reinforced with different types and material of particulates. Eventually, using antimicrobial material could be not feasible from the economic view point especially if used for bulky objects or parts, but antimicrobial material could be efficiently used by using the FFF technology to apply it on the top surface of the object that is potentially subjected to the contamination and this could be optimized using multilayered sandwich components [107,108] to reduce the quantity consumed.

## 5. Conclusions

3D Printing is one the most promising technologies in the field of fabrication and synthesis and has received considerable attention owing to its ability to incorporate antibacterial agents within the structure. Metallic ions, such as copper and silver, are mainly being considered to provide high antibacterial effects in several 3D-developed products. Quaternary ammonium-based salts have also shown high antibacterial potency. The utilization of 3D Printing technology results in faster, compact materials with increased accuracy and enhanced properties as desired. With the increasing rise of 3D Printing in several fields, the potential of is use as an alternative approach is expected. However, because this approach is relatively new, its growth is progressing slowly, and questions remain regarding the reliability of the technique compared with existing well-developed methods that are currently being used.

## Figures and Tables

**Table 1:** Parameters used in selected studies in the 3D printing of antimicrobial material.

Polymer Name	Particle Name	Particle Size	Weight/Volume %	Chemical Agent	Ref
**PCL**	Graphene oxide	Not specified	0%, 5%, and 7.5%,	Not specified	[53]
**Thiol-ene-acrylate**	QUA, SH-QUA	Not specified	2 wt%, 4 wt%, 6, wt%, 8 wt%, 10 wt%	phenylbis(2,4,6-trimethylbenzoyl), phosphineoxide photoinitiator, 1,2,3-benzenetriol	[19]
**stereolithographic resins- UDMA**	Ag-HNT	Nanoparticles	1%, 2%, 5%	triethylene glycol dimethacrylate (TEGDMA), photosensitizer	[55]
**alginate beads**	Copper nanoparticles	Nanoparticles	4 wt% alginate concentration; 50-mM copper salt concentration	Bacterial cellulose	[15]
**PLA**	Copper nanoparticles	Nanoparticles	1% antibacterial nanoparticle additive	-	[58]
**PCL**	Metal ions (Ag, Cu, Zn)	Not specified	Ag-10% *w*/*w* Cu-10% and 25% *w*/*w* Zn-10% *w*/*w*	Tetrahydrofuran (THF) dichloromethane (DCM) -	[42]
**UDMA**	QA_C_n (n=2 to n=16)_pQA_C_12_	Not specified	Nitrogen (N%) linearly related to alkyl chain pQA-C_12_–25 wt%	camphorquinone (CQ); ethyl 4-(dimethylamino)benzoate (EDMAB); glycerol dimethacrylate (GDMA); 2-hydroxyethyl methacrylate (HEMA) for pQA_Cn_12_	2015 [41]

**Table 2:** Printing parameters used in selected studies.

Polymer Name	Nozzle Diameter	Filament Diameter/Printer Settings	Printing Temperature	Filament Process	Tests Done	Reference
**PCL**	xy distances ranged from 200 μm to 400 μm, z-steps from 20 μm to 80 μm, and staggering between layers from 50 μm to 200 μm	Not specified	Not specified	internal diameter of 184 μm (28G) for scaffold plotting	1,4,6,12,16	[53]
**Thiol-ene-acrylate**	Not specified	Not specified	Not specified	Not specified	1,2,9,14	[19]
**stereolithographic resins**	Not specified	Resolution of the device = 50 lm in the Z-direction; exposure time of each layer = 12 s	Not specified	Not specified	1,2,8,5,14,15,18	[55]
**alginate beads**	1.75 mm	3D structures of 30 × 30 × 1 mm^3^ (length × width × height) 1.5 mm of thread spacing; dispensing head temperature of 25 °C; ink extruded with 23-G needle tip at 25 °C; printing speed of 50 mm s^−^^1^; extrusion pressure of 1 bar	25 °C	Not specified	1,5,18	[15]
**PLA**	Not specified	40% infill (hexagon pattern); 50-mm/s print speed; 150–200-mm/s travel speed; 50 °C heated bed; 0.15-mm layer height; 1-mm shell thickness	200 °C	Not specified	1,13	[58]
**PCL**	1.75 mm	- square dressings (20 _ 20-1 mm) for antimicrobial studies and circular dressings (10-mm diameter; 1-mm thickness) −0.1 (mm layer height), with two shells, 100% infill and speed while extruding and while traveling was set to 50 mm/s	170 °C	Extruded/Single screw Ag-80 °C Cu-60 °C Zn-75 °C	1,2,3,4,7,11,17	[41]
**UDMA**	Not specified	z-stage with the substrate moved upward by 200 μm; resolution of the device ~300 μm in the XY-plane and 25 μm in the Z-direction	Not specified	Not specified	1,7,12,14,15,19	[41]

1-Antibacterial testing, 2-TGA, 3-DSC, 4-SEM, 5-TEM, 6-Optical Microscopy, 7-FTIR, 8-EDS, 9-NMR, 10-3D Scanning, 11-3D Scanning, 12-XPS, 13-Box and block test, 14-Mechanical test, 15-Cytotoxicity test, 16-In vivo test, 17-Dissolution test, 18-UV, 19-Contact angle measurements.

**Table 3:** Comparison of antibacterial efficacies from the abovementioned recent studies.

Reinforcement Material	Antimicrobial Synthesis Method	Printing Method	Antimicrobial Activity (%)	Application	Reference
**Graphene oxide**	Dissolution	Wet spinning + AM	80%	Fibrous scaffolds	[53]
**QUA, SH-QUA**	Dissolution (Copolymerization)	DLP	100%	Dental tooth	[19]
**Ag-HNT**	Dissolution in SLR	DLP	99%	Dental composite resin	[55]
**Copper nanoparticles**	Ionic cross-linking	FFF	Not specified	Composite hydrogel	[15]
**Copper nanoparticles**	Purchased PLACTIVE©	Extrusion + FFF	99%	Finger prosthesis	[58]
**Metal ions (Ag, Cu, Zn)**	Hot-melt extrusion	FFF	Not specified	Wound dressing	[41]
**QA_C_n (n=2 to n=16)_ pQA_C_12_**	Dissolution (Copolymerization)	SLA	99%	Dental composite resin	[41]

## Data Availability

The data presented in this study are available on request from the corresponding author.
